# Optimising crown-of-thorns starfish control effort on the Great Barrier Reef

**DOI:** 10.1371/journal.pone.0302616

**Published:** 2025-07-15

**Authors:** Kanupriya Agarwal, Michael Bode, Kate J. Helmstedt

**Affiliations:** 1 School of Mathematical Sciences, Queensland University of Technology, Brisbane, Australia; 2 Centre for Data Science, Queensland University of Technology, Brisbane, Australia; 3 Securing Antarctica’s Environmental Future, Queensland University of Technology, Brisbane, Australia; Central Marine Fisheries Research Institute, INDIA

## Abstract

Outbreaks of crown-of-thorns starfish *Acanthaster planci* (COTS), a disruptive coral-eating predator, are responsible for almost half of total coral cover loss on Australia’s Great Barrier Reef. As the pressures of climate change continue to intensify the frequency and severity of disturbance events such as cyclones and coral bleaching, efficiently managing COTS outbreaks is essential for reef protection. We aim to understand how the spatial distribution and intensity of crown-of-thorns starfish control – specifically manual culling of COTS by human divers – can impact coral cover on the GBR. We construct a metapopulation model based on a predator-prey model with larval dispersal and removal of crown-of-thorns starfish to simulate and compare spatial control strategies. When outbreaks begin on reefs between Cairns and Cooktown, we found the best strategy is to target those reefs at the source of the COTS outbreak. Increasing the spatial spread of control results in a larger spatial area protected across the GBR, but a lower total coral cover on the GBR. Our findings suggest that carefully targeting future control by considering larval connectivity patterns and spatial control strategies could lead to more efficient crown-of-thorns management. With the increasing pressures of climate change, any efficiency gains in reef management will prove beneficial for the Great Barrier Reef.

## 1. Introduction

Coral reefs such as Australia’s Great Barrier Reef (GBR) are among the most diverse ecosystems on Earth, supporting approximately 25% of all marine species in approximately 0.1% of its area [1,2]. Yet, in the 27-year period between 1985 and 2012, the GBR’s coral cover declined by over 50% [3,4]. Outbreaks of crown-of-thorns starfish *Acanthaster planci* (COTS), a disruptive coral-eating predator, are a major driver of coral reef degradation, responsible for 42% of this loss of coral cover on the GBR [3,5]. These corallivorous starfish compound the negative impacts of climate change, including cyclones and coral bleaching [6,7]. Therefore, efficiently managing COTS outbreaks is essential for reef protection, as the pressures of climate change continue to increase the frequency and severity of disturbance events [8].

Managing crown-of-thorns starfish outbreaks is difficult due to the local density of COTS populations on outbreak reefs, and their potential presence across hundreds of reefs and tens of thousands of square kilometres. Indirect actions including marine reserve zoning and improving water quality can impact COTS populations, but the most direct control action is manual culling of individual COTS with lethal injections by human divers [9]. At small local scales, manual control of COTS has varying levels of success [9], since even identifying and detecting COTS can be challenging [10]. However, coordinated large-scale control programs can effectively reduce COTS densities [11,12]. The GBR consists of nearly two thousand connected reefs, so a thorough analysis of COTS control must be considered on a whole-of-GBR scale [13,14]. A range of statistical and simulation models have been used to model crown-of-thorns starfish dynamics across the GBR, but comparative analyses of control strategies are rare [13,15,16]. To undertake a comprehensive analysis of COTS control, we need (1) dynamic large-scale models that can incorporate larval dispersal (integral to coral and COTS population dynamics), (2) models of how COTS control efforts impact densities, and (3) optimisation methods that can compare potential control strategies.

Models of COTS control across the Great Barrier Reef focus on faithfully representing the spatial ecology of outbreaks. For example, the connectivity and larval recruitment of COTS and coral, or on the predator-prey dynamics of COTS outbreaks on individual coral reefs [14,17,18]. These models assume that control efforts will follow current “priority reef” strategies [15,16]. These investigate the effects of current COTS control efforts, however they focus their control in a fixed same spatial area rather than considering the need to spread or focus a fixed control budget over different control area sizes.

We investigate the impact of various crown-of-thorns starfish control strategies on COTS and coral populations on the Great Barrier Reef. We develop a metapopulation model for the GBR, based on Morello et al. [19], which incorporates larval dispersal for both coral and crown-of-thorns starfish, as well as manual culling of crown-of-thorns starfish. We then simulate a crown-of-thorns starfish outbreak for 1705 reefs on the GBR using this metapopulation model. Finally, we simulate various COTS control strategies during an outbreak. We vary the control strategies by varying the spatial spread and intensity of manual culling across the GBR to answer the question: should control intensity be spread or focused?

## 2. Methods

We develop a model which describes the predator-prey interactions between crown-of-thorns starfish (predator) and coral (prey), and incorporates larval dispersal of crown-of-thorns starfish, larval dispersal of coral, and culling of individual crown-of-thorns starfish by human divers at a large number of distinct reefs. The model accounts for area and position (i.e., inside or outside a critical outbreak initiation area) of reefs, and connectivity between them. We use an existing and well-known predator-prey model for a single population from [19] and extend this model to a predator-prey metapopulation model with larval dispersal and crown-of-thorns starfish control. Finally, we parameterise this model for the Great Barrier Reef and various crown-of-thorns starfish control scenarios.

### 2.1 Predator-prey metapopulation model

The model of intermediate complexity from [19] includes a discrete age-structured population model for COTS, a logistic growth model for fast-growing coral, and a logistic growth model for slow-growing coral. We only include fast-growing coral in our model, since these are the preferred food source of COTS; we omit slow-growing coral abundance which has less effect on COTS growth and survival [20]. The age-structured model for COTS has 3 age classes: age 0 (larvae), age 1 (juveniles), and age 2 or older (adults). We extend the discrete time, single reef model in [19] to a metapopulation model using dispersal described in [18], which simulates dispersal between all relevant reefs in the GBR. See S1 Methods for full description of the model.

### 2.2 COTS culling

We model the manual removal of adult COTS as a percentage of the total population at a given reef. In the full metapopulation model (see S1 Methods), a parameter $k_{i,t}$ denotes the proportion of age 2 + COTS that are culled at reef i in year t. These values $k_{i,t}$ will be chosen in our simulations. Here, we are modelling population-dependent culling and assuming that control effort has a linear relationship with the proportion of COTS culled [9,11].

### 2.3 Parameterising the GBR-wide model

Where possible, we use parameter values that were calibrated by Morello et al. [19] for historical coral and COTS populations on the GBR (see Table 1 in S1 Methods). The connectivity matrices for both COTS and coral larval dispersal were simulated by a high-resolution biophysical model of larval connectivity for GBR reefs between 1996–2002 [21]. This biophysical model simulates dispersal for 1705 reefs on the GBR, and thus we were limited to using 1705 reefs in our model, as opposed to the approximately 3000 reefs on the GBR. In 1995, a large outbreak began on the reefs around Lizard Island in the northern GBR; by 2002, the outbreak had moved more than 500 km south, to the reefs between Townsville and Mackay in the central GBR. We use the same connectivity matrices for coral and COTS, a common assumption [18] that overlooks some behavioural and reproductive differences between the organisms. The GBR reef sizes are sourced from the Great Barrier Reef Marine Park Authority [22].

We estimated the proportion of age one COTS that can reproduce using a model from [23] which describes the relationship between COTS age and size, and a model from [24] which describes the relationship between COTS size and gonad weight, which we assume scales linearly with reproductive output (see S1 Methods for more details). Survival rates in the larval and settlement life stages are very difficult to estimate for both COTS and coral. We therefore combined these processes into a survival parameter for each. We chose initial values for these to approximate observed patterns in [19]. Then, we tested the sensitivity of our findings to these parameters and found they had little effect on the numerical simulations, and no effect on the final recommendations (see Figures 4 and 5 in S1 Methods).

We initialise coral cover at 50% on each reef. Although this is higher than the current state of the GBR (where average coral cover ranges between 10–50%, and varies regionally [25]), by simulating a COTS outbreak with abundant coral cover across the GBR, we are better able to discriminate between different control actions.

**Fig 1:**
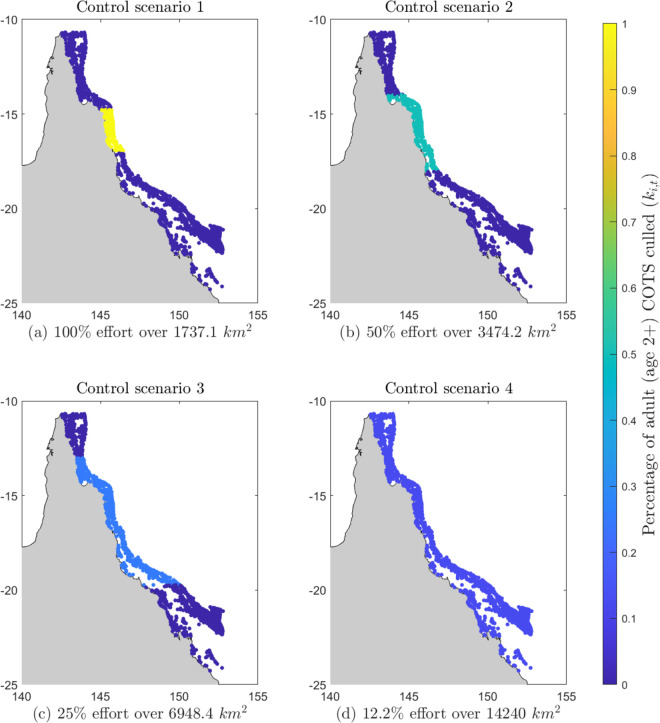
The spatial spread and intensity of COTS control effort across the GBR reefs, for the four scenarios. Each of 1705 individual reefs is indicated by a dot in the map, indicating location but not size. Control effort is quantified by the proportion of adult (age 2+) COTS culled at reef i each year for simulations of 100 years, indicated by colour of the reef (yellow indicating high effort, dark blue indicating low or no effort).


To simulate a COTS outbreak on the GBR, for initial adult COTS populations, we initially place 100 COTS at every reef in the outbreak initiation box (the region between Cairns and Cooktown where COTS outbreaks begin on the GBR; see Figure 1 in S1 Methods) and model the whole GBR for 100 years. To calculate the initial age 1 (denoted $N_{1,i}(0)$) and age 0 COTS (denoted $N_{0,i}(0)$), we recreate a stable age distribution following [19]:


$$N_{0,i}(0)=N_{2,i}(0)e^{2w_{i}(0)M_{s}},$$ (1)

$$N_{1,i}(0)=N_{2,i}(0)e^{w_{i}(0)M_{s}},$$ (2)

where $N_{a,i}(t)$ is the population of COTS of age a at reef i at year 0, $w_{i}(t)$ accounts for the effect of coral abundance on COTS mortality at reef i at time t, and $M_{s}$ is the natural mortality of COTS. This model captures a single outbreak of COTS, including the coral recovery following the outbreak.

### 2.4 Control scenarios

We simulated four different COTS control scenarios, each distributing a fixed control budget across a differently-sized area in the GBR. The simulation was run with and without control for 100 years, to ensure that both short-term and decadal scale consequences become apparent [26]. The four scenarios varied the distribution and intensity of control actions, which remained consistent throughout each simulation (Fig 1). We define the total annual control effort for each scenario as the sum of the control effort at every reef,

$$\text{Total\textbackslash{} Annual\textbackslash{} Effort}=\sum_{i}k_{i,t}\times K_{f,i},$$ (3)

where $k_{i,t}$ is the control effort (measured as a percentage of age 2 + COTS culled at reef i in year t), and $K_{f,i}$ is the carrying capacity of coral cover in square kilometres at reef i, i.e., size of reef i. Here ‘Total Annual Effort’ scales with area, and is measured with units of effort per square kilometre. This value allows for comparison between reefs and strategies, but differs from ‘dive-hours’ used in other COTS literature (e.g., [27]).

Each control scenario has the same total annual effort, distributed differently through space. More vessels used for COTS control result in better outcomes [16], and therefore spreading a fixed budget across a larger area will erode the impact of control at any individual reef. Control science and strategies are rapidly evolving for COTS in the GBR and beyond (Babcock et al., 2020), and so we do not model the control method in detail (see [15,16] for assessment of current control strategies), instead assuming a percentage of the adult COTS can be culled across the area. In scenario 1, we simulate culling 100% of adult COTS, which is equal to $k_{i,t}=1$, at every reef in the initiation box, a total reef area of 1737 $km^{2}$ (Fig 1a). This scenario attempts to halt the outbreak before it spreads beyond the initiation box [28]. In scenario 2, we halve the percentage of COTS culled but double the area controlled, so we cull 50% of adult COTS, or $k_{i,t}$ = 0.5, over a total reef area of 3474.2 $km^{2}$ (Fig 1b). In scenarios 3 and 4, we further increase the number of reefs being treated, each with lower levels of control. By the final scenario, all reefs in the metapopulation are being treated with some control effort. Table 1 describes the details of all four control scenarios including the percentage of adult COTS culled at each reef, the number of reefs controlled, the total area controlled, and the figure which shows the spatial spread of the control effort. We chose the reefs controlled in all scenarios to be centred around the outbreak initiation box since that is the source of the outbreak (Fig 1). We note that areas of live coral cover at reefs may be smaller [29] than the reef sizes obtained from Great Barrier Reef Marine Park Authority [22] and the spatial area controlled in our scenarios (Table 1).

**Table 1:** The details of the four control scenarios simulated using our metapopulation model.

Control scenario	Percentage of adult COTS culled at each reef ($k_{i,t}$)	Number of reefs controlled	Spatial area controlled ($km^{2})$	Spatial spread of reefs controlled
1	100%	163	1737	Fig 1a
2	50%	305	3474	Fig 1b
3	25%	580	6948	Fig 1c
4	12.2%	1705	14240	Fig 1d
Full\ control	100%	1705	14240	Fig 1d

We included an additional control scenario with 100% of COTS culled over the entire GBR. This scenario does not have the same total annual effort as the other strategies described in Table 1, and its effectiveness was not directly compared to the other control scenarios. This scenario is not intended to reflect a realistic control strategy, but is used to establish an upper limit for coral cover increases.

To evaluate the effectiveness of each control strategy, we compare the total area of coral cover across all reefs. This measure assumes that management goals are to maximise average coral cover across the GBR. However, other goals include minimising the number of reefs with low coral cover (e.g., a “minimax” strategy), or maximising the number of reefs with the highest coral cover (“maximin” [30]).

## 3. Results

### 3.1 Crown-of-thorns starfish outbreak dynamics without control

**Fig 2:**
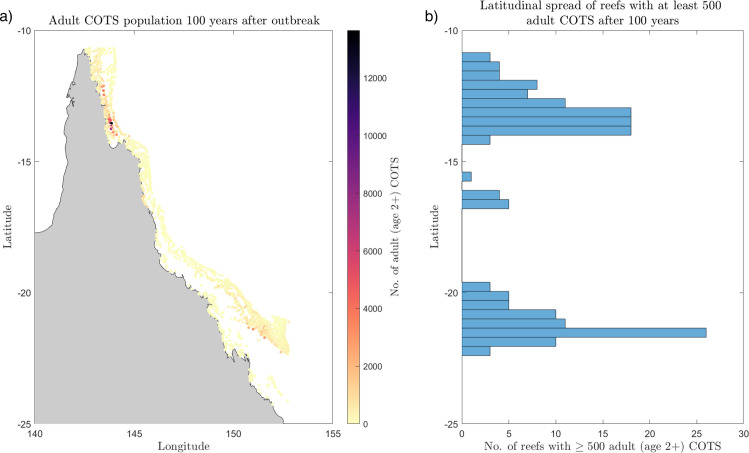
The spatial spread and size of the adult (age 2+) COTS population across the Great Barrier Reef (GBR) after 100 years with no COTS control effort. (a) Map of the GBR, where the colour of each reef corresponds to the number of adult COTS. (b) Latitudinal spread (north-south spread) and count of the reefs on the GBR with at least 500 adult COTS (with a latitudinal bin width of 0.35).

**Fig 3:**
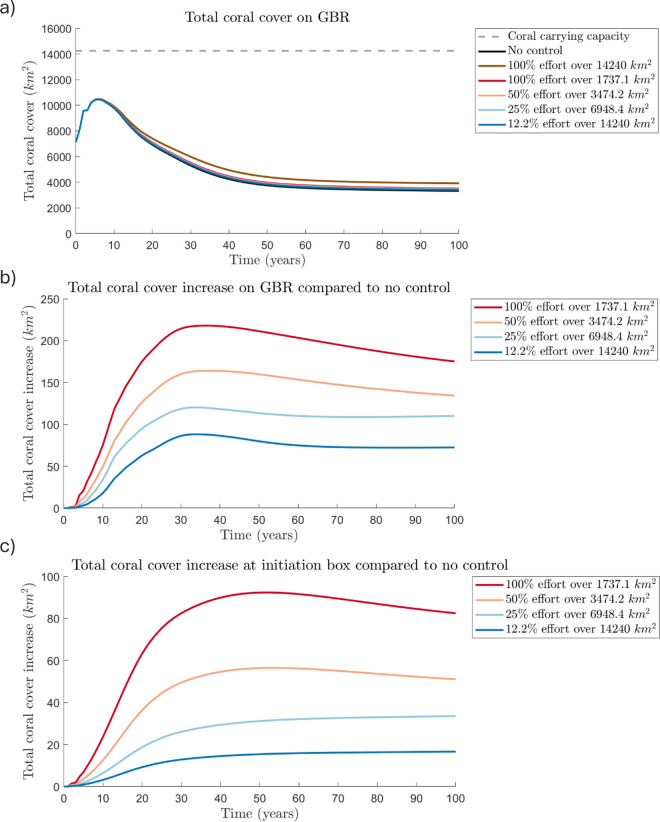
Impacts of COTS control strategies on coral over 100 years. (a) Total coral cover on the GBR, (b) total coral cover increase on the GBR compared to no control effort and (c) total coral cover increase at the initiation box compared to no control effort (in $km^{2}$). Six strategies are shown: 100% control effort over reefs in the initiation box (red), 50% control effort over 3474.2 $km^{2}$ (light red), 25% control effort over 6948.4 $km^{2}$ (light blue), and 12.2% control effort over all reefs (dark blue), no control (black, (a) only), 100% control at all reefs (brown, (a) only). Note here when comparing total coral cover increase, that we are assuming coral cover is 100% of reef area.

Without control, 100 years from the start of the outbreak the simulated crown-of-thorns starfish population spreads to 1700 of the 1705 reefs on the GBR (Fig 2a). However, only 177 reefs achieve high adult COTS populations of 500 or more (Fig 3b). After 100 years, most COTS are not in the outbreak initiation box – only 10 reefs within the outbreak initiation box have 500 or more adult COTS. Instead, the outbreak spreads both north and south on the GBR, and there are two clusters (one north and one south) which have high adult COTS populations (Fig 2). In the north, there is a cluster of 94 reefs with 500 or more adult COTS each, and in the south there is a cluster of 73 reefs with 500 or more adult COTS each. The northern cluster has higher populations of adult COTS – one with 13,649 adult COTS (Fig 2a, darkest purple).

The solution to our model exhibits travelling waves, moving both southward and northward from the initiation box (Figure 2 in S1 Methods). This agrees in part with the general phenomenon observed on the GBR since 1985: a series of southward travelling waves (1985–1995, 1998–2008, 2012–2018 [31]). COTS density data does not show outbreak waves travelling north from the initiation box [31], as predicted by our model, but systematic observations of COTS densities north of latitude 12 south are rare before 2012 [31,32].

Despite the partial qualitative agreement on southward travelling waves, the model does not reproduce the speed of COTS outbreak progression on the GBR, nor their recurrence. COTS outbreaks tend to reach the southern GBR within 10–15 years [28]. By contrast, our model predicts a slower progression, with outbreaks reaching latitude 20 south after 25–35 years. Moreover, the model does not predict any subsequent outbreaks in the initiation box over the modelled 100-year timeframe. Instead, COTS abundance reaches a stable equilibrium across the metapopulation.

### 3.2 Comparison of crown-of-thorns starfish control scenarios

The total coral cover on the GBR follows the same general trend over 100 years for all scenarios simulated, including for no control effort (Fig 3a). Coral cover increases initially, while the COTS population is still relatively small and localised, decreases for approximately 50 years as the outbreak spreads through the GBR, and then stabilises for the last 40–50 years, with COTS becoming endemic. A net reduction of coral cover after 100 years is observed in all control scenarios – even culling 100% of COTS across all reefs (Fig 3a). However in comparison to a scenario with no control, the four control scenarios with the same total annual effort achieve modest increases in total coral cover at the end of the 100 years (between 72 km^2^ and 175.2 km^2^ additional live coral cover – assuming that coral cover is 100% of reef area) (Fig 3b). We see that culling a higher percentage of COTS over a smaller area is more effective at increasing the total coral cover on the GBR, compared to no control, than culling a smaller percentage of COTS over a larger area. Here we examine a 100-year horizon to observe the long-run behaviour of the system, but note that most of the long-term benefit is achieved by year 30 (Fig 3a–3c).

The most effective control strategy is to cull 100% of adult COTS over 1737 $km^{2}$ (or the initiation box) which increases total coral cover on the GBR by 175 $km^{2}$ (assuming that coral cover is 100% of reef area) compared with no control after 100 years. The least effective strategy, controlling every reef on the GBR with 12.2% effort is only able to increase total coral cover on the GBR by 72 $km^{2}$ (assuming that coral cover is 100% of reef area) after 100 years. Thus, the most effective control scenario can protect approximately 2.4 times more coral cover across the GBR than the least effective scenario after 100 years (if 100% effectiveness were possible). Coral cover within the initiation box mirrors these trends, where the most effective scenario at increasing total coral cover is targeting the initiation box with 100% effort (Fig 3c).

### 3.3 Spatial spread and intensity of coral cover increase on the GBR

**Fig 4:**
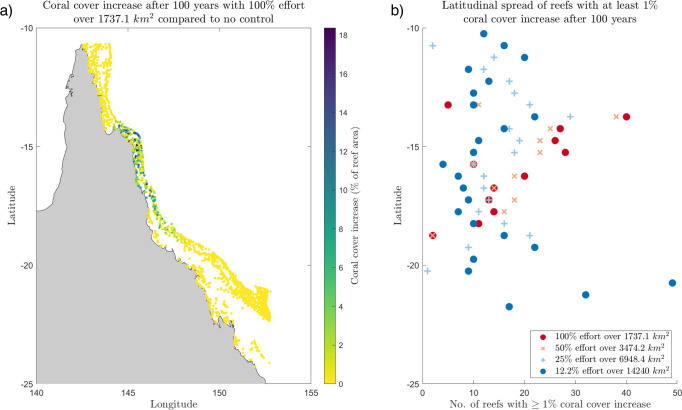
Spatial impacts on coral cover from COTS control strategies 100 years after outbreak. (a) Map of coral cover increase on the GBR for 50% effort over 3474.2 $km^{2}$ compared to no control. Colour of each reef corresponds to the increase in coral cover, as a percentage of reef area. (b) Latitudinal spread (north-south spread) of reefs with at least 1% increase in coral cover compared to no control for control scenarios: 100% effort over 1737.1 $km^{2}$ reefs (dark red dots), 50% effort over 3474.2 $km^{2}$ (light red crosses), 25% effort over 6948.4 $km^{2}$ (light blue plus signs), and 12.2% effort over 14240 $km^{2}$ (dark blue dots).

Culling 50% of adult COTS over 3474 $km^{2}$ (including the area of the initiation box) is a more effective and plausible strategy if the goal is to increase the total coral cover across the GBR. This control strategy increases coral cover on 99% of the reefs on the GBR compared to no control, or on 1696 of the 1705 reefs, by up to 11% of the reef area (Fig 4a). However, on 1487 of these reefs, the increase in coral cover is less than 1% of the reef area. The remaining 209 reefs (or 12% of the number of reefs on the GBR) see an increase in coral cover of 1% or more over a no-control strategy, and are mainly located on the reefs within and surrounding the outbreak initiation box, where the control effort is located (Fig 4b). Thus, although the 50% effort over 3474 $km^{2}$ scenario is more effective at increasing total coral cover on the GBR, the spatial extent of coral cover increase is limited to the mid-region reefs of the GBR.

Increasing the spatial spread of control increases the spatial extent of reefs with at least 1% increase in coral cover after 100 years over a no-control strategy (Fig 4b). Thus, spreading the control effort wider on the GBR results in a lower increase in total coral cover compared to no control, but a higher latitudinal spread of reefs with a high increase in coral cover. If the goal is to protect coral cover across a larger spatial area on the GBR, then the most effective control strategy is to cull over the entire area of the GBR. With this control strategy, 350 reefs (or 21%) of the reefs on the GBR see an increase in coral cover of 1% or higher over a no-control strategy, but the largest increase in coral cover at a single reef is only 4%.

**Fig 5:**
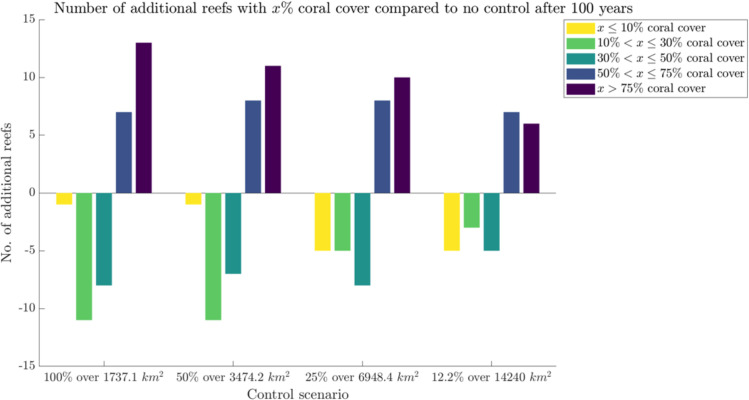
Number of reefs with different coral covers compared to no control scenario. The number of additional reefs with x% coral cover for the various control scenarios compared to no control effort, where the colour of the bar corresponds to the reef’s coral cover, as a percentage of reef area: reefs with x > 75% coral cover (dark purple), reefs with 50% < x ≤ 75% coral cover (dark blue), reefs with 30% < x ≤ 50% coral cover (light blue), reefs with 10% < x ≤ 30% coral cover (green), and reefs with x ≤ 10% coral cover (yellow). The x-axis shows the four control scenarios: 100% effort over 1737.1 $km^{2}$ reefs, 50% effort over 3474.2 $km^{2}$, 25% effort over 6948.4 $km^{2}$, and 12.2% effort over 14240 $km^{2}$ (or the entire GBR).

The choice of control strategy varies when considering different optimisation objectives. When minimising the number of reefs with 10% coral cover or less – reefs classified as ‘low coral cover’ by the Australian Institute of Marine Sciences [25] – both 25% effort over 6948 $km^{2}$, and 12.2% effort over 14240 $km^{2}$ (or the entire GBR) are top performers (minimising the value of the yellow bar, Fig 5) as there are 5 fewer reefs in this category compared to no control. Alternatively, if we were to apply the maximin strategy and maximise the number of reefs with greater than 75% coral cover (maximising the value of the purple bar, Fig 5), the most effective control strategy would be 100% effort over 1737 $km^{2}$ as there are 13 additional reefs within this category compared to no control.

## 4. Discussion

To investigate the impact of various COTS control strategies on GBR coral coverage, we developed a predator-prey metacommunity model based on [19], and simulated a COTS outbreak and a control response. We found that the most effective strategy was to target the source of the COTS outbreak, i.e., to cull as many adult COTS as possible within the initiation box. This strategy was the most effective at increasing total coral cover (compared to no control), with an increase of 175 $km^{2}$ of live coral (assuming that coral cover is 100% of reef area) after 100 years. Regardless of the outbreak size, targeting the source of the COTS outbreak will be beneficial for total coral cover on the GBR.

We also found that increasing the spatial extent of control effort delivered benefits to a wider spatial area, but a lower total increase compared to no control. There is therefore a trade-off between increasing the total coral cover and expanding the range of reefs that benefit. So, if the goal is to protect a larger spatial area of coral cover across the GBR then spreading the control effort is more effective. However, if the goal is to increase total coral cover on the GBR then targeting the source of the outbreak is more effective.

The finding that highest total coral cover benefit is achieved by ensuring effective control on a limited area rather than partial control over a larger area is consistent with the guiding principles of integrated pest management. An Integrated Pest Management study for COTS identified the need to reduce the spread of an outbreak and focus prevention efforts on the initiation box [33]. Testing these recommendations has resulted in improved management at a reef-scale [34]. Our findings both support these recommendations and represent a step toward their identified need for regional-scale optimisation of COTS control.

Our simulated outbreaks spread both north and south from the initiation box, a result that diverges from the standard belief that COTS outbreaks spread south from this area [14,35]. However, historical survey effort for COTS is low – often completely absent – on reefs north of Lizard Island, and more recent surveys are discovering unexpected dynamics in the Far Northern Management section of the GBR [31]. As a consequence, it is possible that outbreaks begin north of the “initiation box”, or that outbreaks move north as well as south. We also only simulate outbreaks that begin in the initiation box, which overlooks the potential role of southern reefs (particularly in the Swains group) as both a reservoir for COTS populations, and as a potential independent source of outbreaks [31]. While our model captures coral recovery and sustained moderate levels of COTS following an outbreak (see Figure 3 in S1 Methods), it is not designed to represent multiple waves of COTS outbreaks from the initiation box.

Our model is not a predictive model, and the aim is not to predict the absolute size of COTS or coral populations into the future for the given control strategies. As with any large-scale ecosystem models, we needed to make a range of assumptions when parameterising the ecological elements of the model. Our model captures the predator-prey dynamics between coral and COTS that drive outbreak dynamics, but does not consider any of the environmental factors that are also thought to play important roles (e.g., environmental triggers [36]), nor the species that are thought to predate COTS [37]. Moreover, we did not considered interacting factors that could alter the strength of the effects of COTS on coral populations, such as heterogenous fishing pressures, nearby urbanization [38], water quality [39], or environmental forcing like cyclones and bleaching [26]. Our larval connectivity parameters were based on high-resolution biophysical modelling, but assumed the same dynamics for both COTS and corals. While this is a common assumption (e.g., [18]), their dispersal dynamics are known to differ in larval behaviour and ontogeny (e.g., their pelagic larval duration, buoyancy [40]).

We also made a range of simplifications to our model of COTS management. Firstly, the control scenarios simulated using our model are simplistic; although we identified the best strategy from our four control scenarios, this is not necessarily the optimal control strategy for the GBR, since we did not search through the very large number of alternative spatiotemporal strategies. Rather, we have attempted to explore how spreading the spatial extent of COTS control can affect coral and COTS populations on the GBR and found that this can have a significant impact on the populations, particularly on the spatial spread of populations.

As with any large-scale ecosystem models, we needed to make a range of assumptions when parameterising this model. Firstly, the control scenarios simulated using our model are simplistic. The cost of culling COTS does not increase linearly with the number of COTS culled; the less COTS there are at a reef, the more difficult it is to find and cull COTS. The cost of culling large proportions of COTS populations will vary from reef to reef, depending on the population size and habitat at that reef. There are also many costs associated with sending human divers to reefs, and thus the location of each reef will also have an impact on the cost; reefs that are harder to get to will be more expensive to cull at. It is also infeasible to be culling at the upper range of control reefs we have simulated; there is currently only sufficient funding to control a few hundred reefs in any given year [12]. However, by simulating the extremes of culling, our results range across the scope of potential actions. Furthermore, in our model we only incorporate culling of adult COTS, but in reality culling will target multiple size classes. We assume that adult culling occurs after they reproduce. This has a major impact on our results: even when we culled 100% of adults, all were allowed to reproduce first, leading to higher average populations. In reality, when culling sometimes the adult COTS will have reproduced already and other times not [41].

The spatial distribution of COTS control can strongly impact the amount and extent of coral cover on the GBR, and is therefore an important factor when choosing control strategies. Any efficiency gains in COTS control will prove to be beneficial to the coral health and biodiversity of the GBR, particularly as pressures from climate change accelerate throughout the century. These results align with common recommendations in other invasive species management scenarios, suggesting the general approach to focus eradication efforts over a carefully targeted small areas can be an effective and efficient use of limited resources.

## Supporting information

S1 Methods Supplementary methods. (DOCX)

S1 File Matlab code. (ZIP)
