# Supplementary material for: Optimising crown-of-thorns starfish control effort on the Great Barrier Reef
Source: PLoS One. 2025 Jul 15;20(7):e0302616. doi: 10.1371/journal.pone.0302616 (PMC12262835; doi:10.1371/journal.pone.0302616)
Supplement: S1 Methods — (DOCX) [file pone.0302616.s001.docx]

**Supplementary materials for Agarwal et al.**

1. **Extended MICE model of crown-of-thorns starfish and coral on the Great Barrier Reef.**

The predator-prey metapopulation model equations for coral and COTS are as follows:

$$N_{0,i}\left( t+1 \right)=\tau_{i}\left( t+1 \right)$$

$$N_{1,i}\left( t+1 \right)=N_{0,i}\left( t \right) e^{-w_{i}\left( t \right) M_{s}}$$

$$N_{2,i}\left( t+1 \right)=\left( N_{1,i}\left( t \right)+\left( 1-k_{i,t} \right) N_{2,i}\left( t \right) \right)e^{-w_{i}\left( t \right) M_{s}}$$

$$C_{i}\left( t+1 \right)=C_{i}\left( t \right)+r_{f} C_{i}\left( t \right)\left( 1-\frac{C_{i}\left( t \right)}{K_{f,i}} \right)-Q_{i}\left( t \right)+\sigma_{i}\left( t+1 \right)$$

Eq. (1) – Eq. (4)

where,

$$w_{i}\left( t \right)=1-\alpha_{fs}\left( \frac{C_{i}\left( t \right)}{1+C_{i}\left( t \right)} \right)$$

$$Q_{i}\left( t \right)=\left( 1-\rho_{i}\left( t \right) \right)\frac{\alpha_{sf,i}\left( N_{1,i}\left( t \right)+N_{2,i}\left( t \right) \right)C_{i}(t)}{1+\exp\left( -\frac{(N_{1,i}\left( t \right)+N_{2,i}\left( t \right))}{\beta_{sf,i}} \right)}$$

$$\rho_{i}\left( t \right)=\exp\left( -\frac{\alpha_{f} C_{i}\left( t \right)}{K_{f,i}} \right)$$

$$\tau_{i}\left( t+1 \right)=\sum_{j} V_{s} \left[ \Omega_{s} \right]_{ji}\left( \mu_{s} N_{1,j}\left( t \right)+N_{2,j}\left( t \right) \right)\lambda_{s}$$

$$\sigma_{i}\left( t+1 \right)=\sum_{j} {V_{f} \left[ \Omega_{f} \right]}_{ji} C_{j}(t) \lambda_{f}.$$

Eq. (5) – Eq. (9)

Here $N_{a,i}(t)$ denotes the number of COTS of age $a=0, 1, 2$, at reef $i$ in year $t$ (note $N_{2,i}(t)$ combines all COTS aged over 2). Equations 1 – 3 describe the age-structured population model for COTS with population growth (a function of coral abundance, Equation 5), demographic mortality, and mortality from manual culling. Equation 1, describing COTS of age 0, relies upon connectivity with other reefs in the metapopulation for reproduction via dispersal. Fast-growing coral cover at reef $i$ in year $t+1$, $C_{i}(t+1)$, is modelled in square kilometres (Equation 4) and incorporates demographic growth and mortality, consumption by COTS (via Equation 6), and reproduction via dispersal. Larval recruitment for both COTS (Equation 8) and coral (Equation 9) are driven by connectivity matrices $\Omega_{s}$ and $\Omega_{f}$ respectively, the per-capita fecunditiy of each, and survival rates after settlement.

See Table 1 for the full list of variables used in this model. In this model, we assume larval recruitment (for both coral and COTS) and coral consumption, occurs first, then adult COTS culling occurs, and finally coral growth and natural mortality of adult and juvenile COTS occurs.

*Table 1: Variables used in the mathematical models with the description, units, values, and the data source.*

| **Variable** | **Description** | **Units** | **Value** | **Data Source** |
| --- | --- | --- | --- | --- |
| $s$ | Index denoting crown-of-thorns starfish (also referred to as COTS) | n/a | n/a | n/a |
| $f$ | Index denoting coral or fast-growing coral | n/a | n/a | n/a |
| $N_{0,i}(t)$ | Number of COTS of age 0 (larvae) at reef $i$ at the start of year $t$ | Individuals | Given by Eq. (10) | n/a |
| $N_{1,i}(t)$ | Number of COTS of age 1 (juveniles) at reef $i$ at the start of year $t$ | Individuals | Given by Eq. (11) | Modified from Morello et al. (2014) |
| $N_{2,i}(t)$ | Number of COTS of age 2 or older (adults) at reef $i$ at the start of year $t$ | Individuals | Initial value of 100 at every reef within initiation box and 0 at all other reefs | Modified from Morello et al. (2014) |
| $C_{i}(t)$ | Coral cover, in square kilometres, at reef $i$ at the start of year $t$ | $km^{2}$ | Initial value of 50% of carrying capacity | Obtained from Great Barrier Reef Marine Park Authority (1998) |
| $Q_{i}(t)$ | Coral cover consumed by COTS, in square kilometres, at reef $i$ in year $t$ | $km^{2}$ | Function (no initial value) | n/a |
| $\rho_{i}(t)$ | Accounts for proportion of coral consumed by COTS at reef $i$ in year $t$ | None | Function (no initial value) | n/a |
| $w_{i}(t)$ | Accounts for effect of coral abundance on COTS mortality at reef $i$ in year $t$ | None | Function (no initial value) | n/a |
| $r_{f}$ | Intrinsic growth rate of coral | Per year | 0.5/year | Estimated by Morello et al. (2014) |
| $K_{f,i}$ | Carrying capacity of coral cover, in square kilometres, at reef $i$ i.e., size of reef $i$ | $km^{2}$ | See Supporting Information | Obtained from Great Barrier Reef Marine Park Authority (1998) |
| $M_{s}$ | Natural mortality of COTS | Exponential constant rate | 2.56 | Estimated by Morello et al. (2014) |
| $\alpha_{f}$ | Parameter which scales the proportion of coral cover to account for coral consumed by COTS | Dimensionless rate (none) | 5 | Estimated by Morello et al. (2014) |
| $\alpha_{sf,i}$ | Effect of COTS on coral at reef $i$ | Dimensionless rate (none) | See Supporting Information | Estimated by Morello et al. (2014) |
| $\beta_{sf,i}$ | Effect of COTS on coral at reef $i$ | Dimensionless rate (none) | See Supporting Information | Estimated by Morello et al. (2014) |
| $\alpha_{fs}$ | Effect of coral on COTS | Dimensionless rate (none) | 0.258 | Estimated by Morello et al. (2014) |
| $\tau_{i}\left( t \right)$ | Number of age 0 COTS (larvae) settling at reef $i$ in year $t$ | Individuals | Function (no initial value) | n/a |
| $\sigma_{i}\left( t \right)$ | Coral cover at reef $i$ in year $t$, in square kilometres, which grows from coral larvae that arrive at reef $i$ in year $t-1$ | $km^{2}$ | Function (no initial value) | n/a |
| $\Omega_{s}$ | Connectivity matrix for COTS larval dispersal where the element $\left[ Ω_{s} \right]_{ji}$ is the percentage of COTS larvae born at reef $j$ that disperses to reef $i$ | Percentage (none) | See Supporting Information | Obtained from Bode et al. (2012) |
| $\Omega_{f}$ | Connectivity matrix for coral larval dispersal where the element $\left[ Ω_{f} \right]_{ji}$ is the percentage of coral larvae born at reef $j$ that disperses to reef $i$ | Percentage (none) | See Supporting Information | Obtained from Bode et al. (2012) |
| $V_{s}$ | Percentage of COTS larvae that survive once they settle at a reef | Percentage (none) | 0.25 or 25% | n/a |
| $V_{f}$ | Percentage of coral larvae that survive once they settle at a reef | Percentage (none) | 0.9 or 90% | n/a |
| $\mu_{s}$ | Proportion of age 1 COTS that are able to reproduce every year | Percentage (none) | 0.1512 or 15.12% | Estimated using Lucas (1998) and Babcock et al. (2016) |
| $\lambda_{s}$ | COTS fecundity i.e. number of COTS larvae (age 0 COTS) produced by each adult (age 2+) COTS every year | Individuals | 5000 | n/a |
| $\lambda_{f}$ | Coral fecundity i.e. coral cover produced every year, as a proportion of coral cover at a given reef | Dimensionless rate (none) | 0.1 | n/a |
| $k_{i,t}$ | Control effort i.e. percentage of age 2+ COTS culled at reef i in year $t$ | Percentage (none) | $k_{i,t}\in\left( 0,1 \right)$ where  1 = 100% | Chosen based on control scenario |

1. **Components of the extended MICE model of crown-of-thorns starfish and coral on the Great Barrier Reef.**

2.1.1 Predator model

Equations 1 – 3 describe the age-structured population model for COTS. $N_{a,i}(t)$ denotes the number of COTS of age $a=0, 1, 2$, at reef $i$ in year $t$ (note $N_{2,i}(t)$ combines all COTS aged over 2). Equation 1 is the number of COTS larvae (age 0) at reef $i$ in year $t+1$, ($N_{0,i}(t+1)$), and is equal to $\tau_{i}(t+1)$, or the number of COTS larvae arriving at reef $i$ in year $t+1$. Equation 2 describes the number of age 1 (juvenile) at reef $i$ in year $t+1$ ($N_{1,i}\left( t+1 \right)$) is equal to the number of age 0 COTS at reef $i$ in the previous year $t$ ($N_{0,i}\left( t \right)$), multiplied by an exponential term ($e^{-w_{i}\left( t \right)M_{s}}$) which accounts for COTS mortality. Age 2+ COTS (Equation 3) are modelled very similarly to age 1 COTS, except the number of age 2+ COTS in the current year ($N_{2,i}\left( t+1 \right)$) is equal to the number of age 1 COTS at reef $i$ last year ($N_{1,i}\left( t \right)$) plus the number of age 2+ COTS at reef $i$ last year after manual culling ($(1-k_{i,t})N_{2,i}\left( t \right)$), multiplied by the same exponential term ($e^{-w_{i}\left( t \right)M_{s}}$). Note that $k_{i,t}$denotes the percentage of age 2+ COTS that are culled at reef $i$ in year $t$. The function $w_{i}(t)$ in the exponential terms, given by Equation 5, accounts for the effect that coral abundance has on COTS mortality i.e. when there is low coral abundance, the COTS population will decrease due to a diminished food source and when there is high coral abundance, the COTS population will increase due to the abundant food source. The other term in the exponential is the variable $M_{s}$, which is the natural mortality rate of COTS.

2.1.2 Prey model

Fast-growing coral cover at reef $i$ in year $t+1$, $C_{i}(t+1)$, is modelled in square kilometres, and is given by Equation 4. The coral cover at reef $i$ in year $t+1$ ($C_{i}(t+ 1)$) is the coral cover at reef $i$ in the previous year $t$ ($C_{i}(t)$), plus the logistic growth equation to account for natural growth and death of coral, minus the coral cover consumed by COTS at reef $i$ in the previous year $t$ ($Q_{i}(t)$) and plus the coral cover which grows at reef $i$ from the previous year’s coral larvae ($\sigma_{i}(t+1)$). The logistic growth equation for coral accounts for the population dynamics of coral, where $r_{f}$ is the intrinsic growth rate of coral and $K_{f,i}$ is the carrying capacity of coral cover of reef $i$.

The third term in Equation 4 is the coral cover consumed by COTS, in $km^{2}$, at reef $i$ in year $t$, $Q_{i}(t)$, and is given by Equation 6. Note here that $Q_{i}(t)$ accounts for both age 1 and age 2+ COTS feeding on coral. The COTS population terms in Equation 6, ($(N_{1,i}(t)+N_{2,i}(t))/(1 + e^{-(N_{1,i}\left( t \right)+N_{2,i}\left( t \right))})$), describes how the COTS population affects the coral consumption; when there are more COTS, there will be higher levels of coral consumption. The parameters, $\alpha_{sf,i}$ and $\beta_{sf,i}$ in Equation 6, also account for the negative effect that COTS have on coral, by scaling the COTS population size by a linear factor. The term for coral cover, $C_{i}(t)$ is also on the numerator of the fraction in Equation 6 since the more coral cover there is, the more COTS there will be and thus the more coral consumption there will be. Finally, the term, $1-\rho_{i}(t)$ in Equation 6 accounts for the proportion of coral cover consumed by COTS, based on the coral cover present. The value of $\rho_{i}(t)$, given by Equation 7, will vary between 0 and 1 and is based on the available food supply of fast-growing coral at reef $i$ in year $t$ compared to the carrying capacity of coral. The parameter $\alpha_{f}$ in Equation 7 scales the proportion of coral cover present to more accurately account for the proportion of coral cover consumed by COTS.

2.1.3 Larval recruitment

Equation 8 describes the number of COTS larvae (age 0) arriving at reef $i$ in year $t+1$, and is denoted by $\tau_{i}(t+1$). COTS larval recruitment at reef $i$ is modelled as the sum of the larvae arriving at reef $i$ from every other reef $j$ (including reef $i$). In Equation 8, $\lambda_{s}$ is the per capita COTS fecundity or the number of COTS larvae produced by each adult COTS every year, $V_{s}$ is the proportion of COTS larvae that survive once they arrive at a reef, and $Ω_{s}$ is the connectivity matrix for COTS larval dispersal, where$\left[ Ω_{s} \right]_{ji}$ is the proportion of COTS larvae from reef $j$ that disperse to reef $i$. So, we calculate the number of COTS larvae arriving at reef $i$ from a single reef $j$ by multiplying the number of COTS that can reproduce at reef $j$ (all of the adult COTS and some proportion, $\mu_{s}$, of age 1 COTS), $N_{2,j}\left( t \right)+\mu_{s}N_{1,j}(t)$, with $\lambda_{s}$ to give the total number of COTS larvae produced at reef $j$. This is then multiplied by the percentage of larvae at reef $j$ which disperse to reef $i$ and survive, $V_{s}\left[ Ω_{s} \right]_{ji}$. We then sum over all reefs $j$ to find the COTS larvae the settle at reef $i$, $\tau_{i}(t+1)$. For any given reef the total percentage of COTS larvae that disperse to other reefs will not necessarily add up to 100%. It is more likely to be a number less than 100%, since some percentage of larvae will disperse into the ocean and never end up at a reef i.e., the larvae will die. This COTS larval mortality is accounted for through the connectivity matrix, where we assume that from the larvae born at reef $j$, the total percentage of larvae that arrive at every other reef $i$ (including reef $j$) must be less than or equal to 1.

Coral larval recruitment is modelled very similarly to COTS larval recruitment and is described by Equation 9, where $\sigma_{i}(t+1)$ is the coral cover at reef $i$ in year $t+1$, in square kilometres, which grows from coral larvae that arrive at reef $i$ in year $t$. Here, $\lambda_{f}$ is the per capita coral fecundity or the coral cover produced every year as a proportion of coral cover at a given reef, $V_{f}$ is the proportion of coral larvae that survive once they arrive at a reef, and $Ω_{f}$ is the connectivity matrix for coral larval dispersal, where $\left[ Ω_{f} \right]_{ji}$is the percentage of coral larvae from reef $j$ that disperse to reef $i$. To account for coral larval mortality, we assume that in the coral connectivity matrix, from the larvae born at reef $j$, the total percentage of larvae that arrive at every other reef $i$ (including reef $j$) must be less than or equal to 1. Coral larval survival is accounted for in our model via the logistic growth model for coral in Equation 4.

1. **Initiation box**

The coordinates for the vertices of the initiation box are 14.75°S - 17°S, 145°E - 147°E, and encompass 163 individual reefs in total (Reichelt et al., 1990; Vanhatalo et al., 2017).


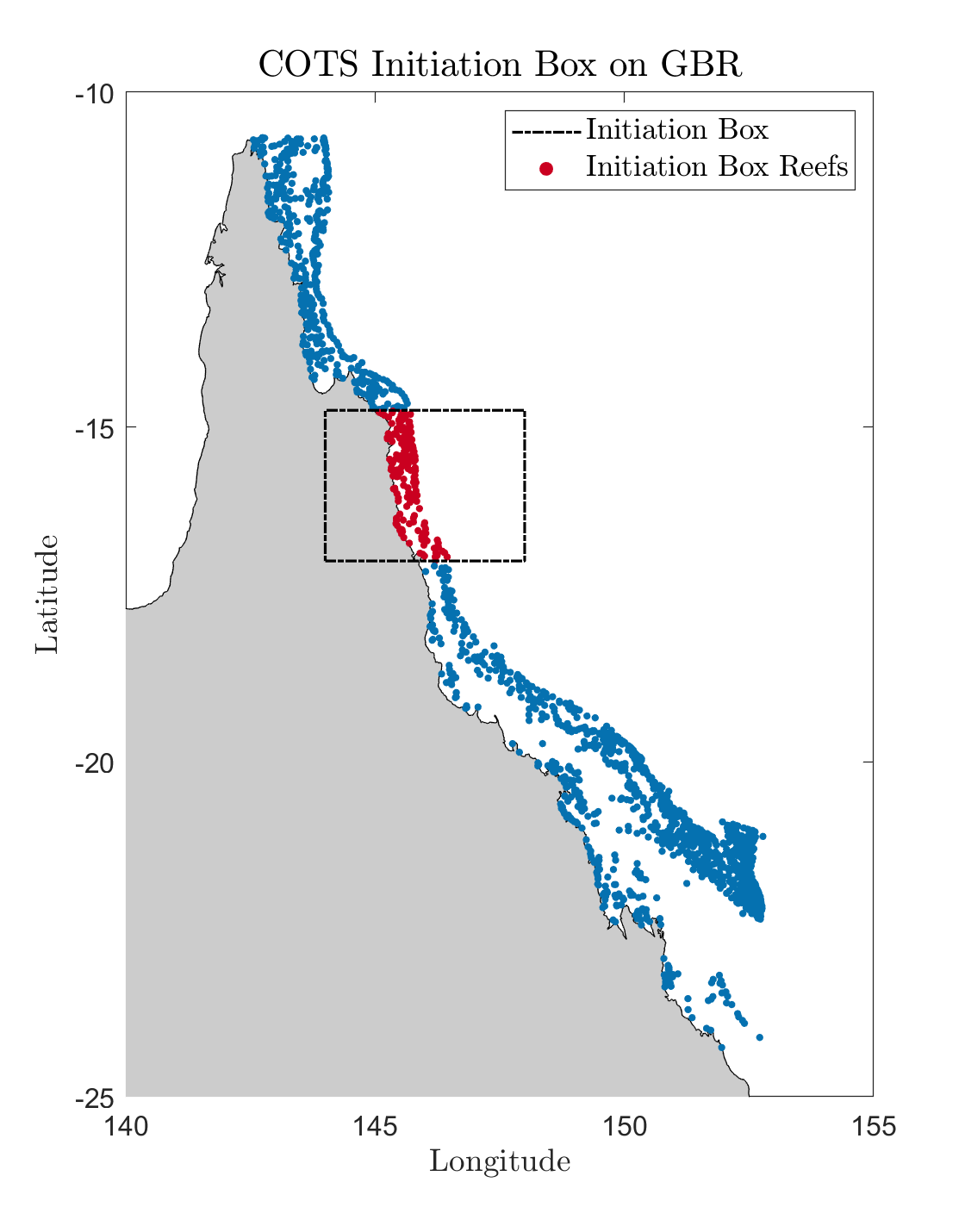


Figure 1: Location of the COTS initiation box (dashed line) enclosing 163 reefs (red dots) on the GBR., with coordinates 14.75°S - 17°S, 145°E - 147°E.

1. **Wave dynamics of COTS spread**

**
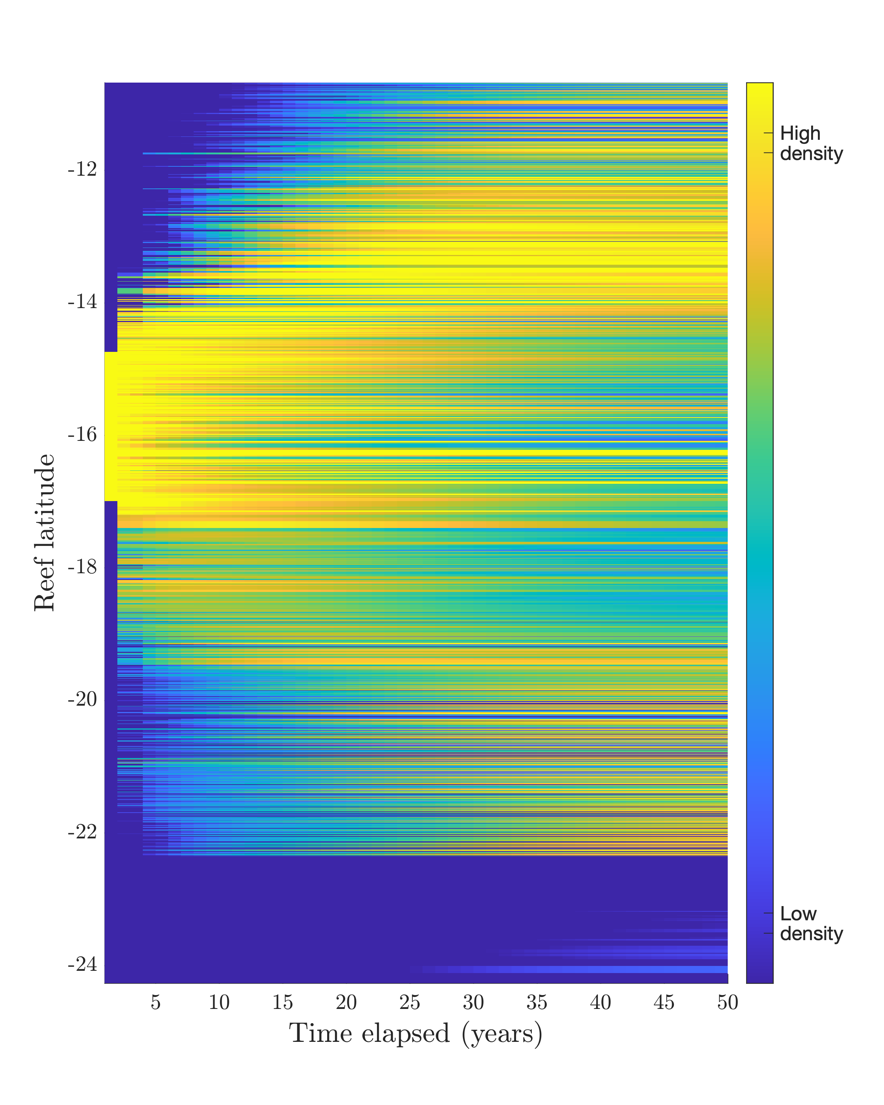
**

Figure 2 Density of COTS over a 50 year outbreak (time on the x axis) across the latitudes of the Great Barrier Reef (latitude on the y axis). Colour intensity in a pixel at a given time and latitude gives the average density across the strip of that latitude at that time in a modelled outbreak. Yellow indicates a high density of COTS, green moderate, dark blue very low. Tracking the yellow sections from left to right illustrates the movement of a high-density patch north and south through time.


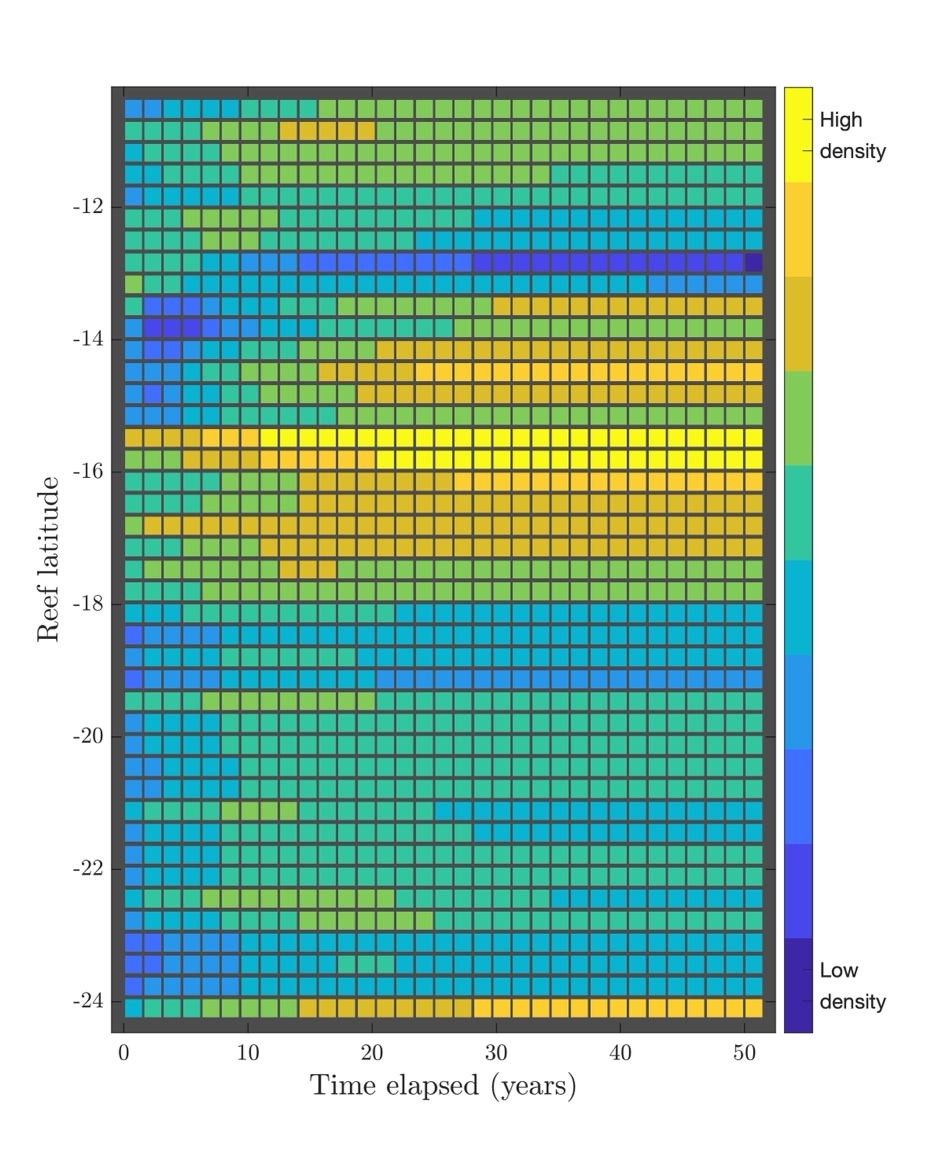


Figure 3 Density of coral over a 50 year outbreak (time on the x axis) across the latitudes of the Great Barrier Reef (latitude on the y axis). Colour intensity in a pixel at a given time and latitude gives the average density across the strip of that latitude at that time in a modelled outbreak. Yellow indicates a high density of coral, green moderate, dark blue very low. Tracking the yellow sections from left to right illustrates coral recovery following COTS outbreak over time.

1. **Parameterising larval survival rates**

We chose initial values for the survival rate of larvae of COTS and coral on new reefs after dispersal to approximate observed patterns in Morello *et al.* (2014). Then, we tested the sensitivity of our findings to these parameters and found they had little effect on the numerical simulations, and no effect on the final recommendations (Figs 4, 5).


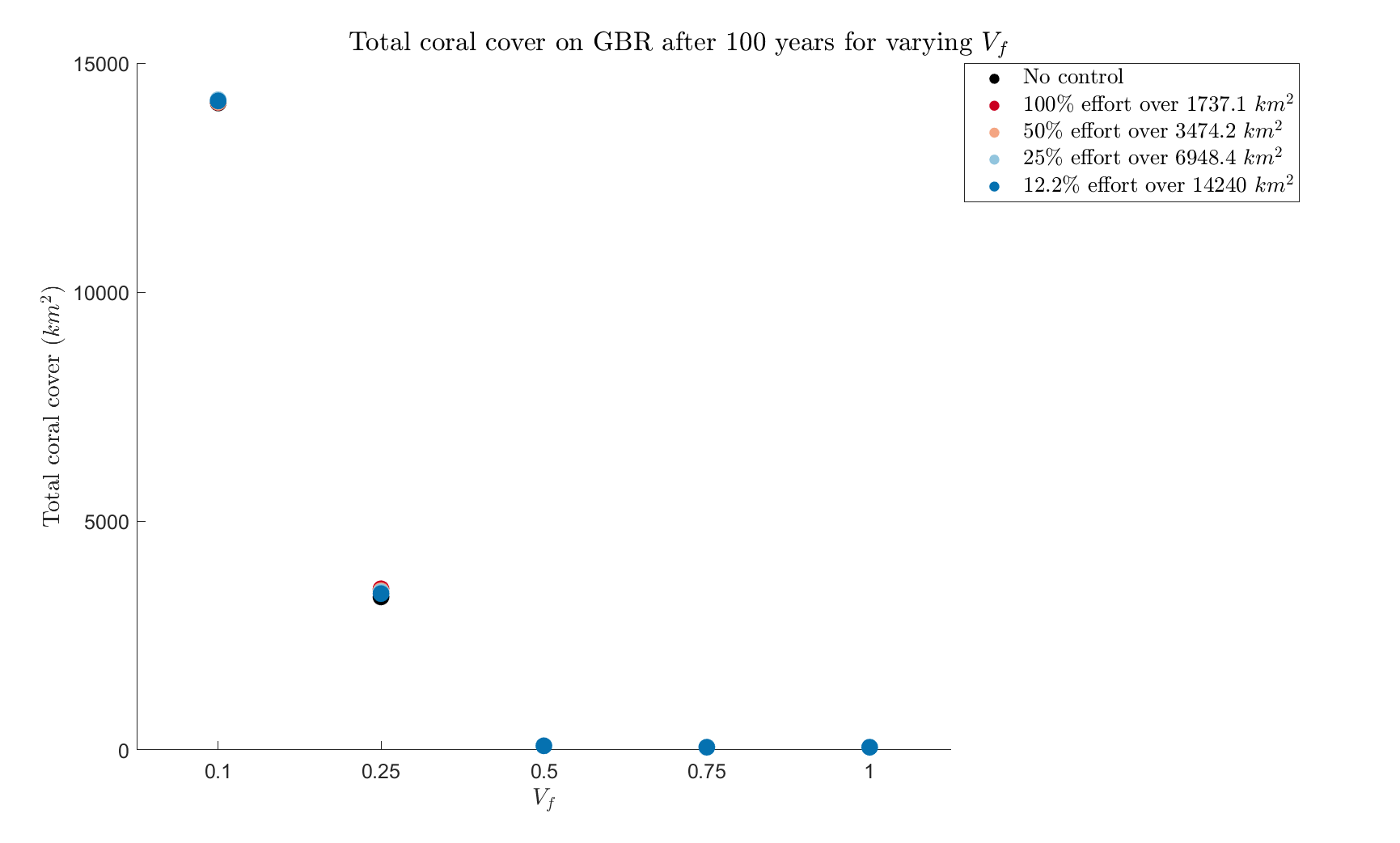


Figure 4 Total coral cover in km2 (sum of coral cover at each reef) on the Great Barrier Reef (GBR) after 100 years, for varying values of V_f, the percentage of coral larvae that survive once they settle at a reef, and varying control scenarios. The numbers tested for V_f are: 0.1, 0.25, 0.5, 0.75, and 1, and are shown on the x-axis. The control scenarios are: no control (black dots), 100% effort over 1737.1 km2 (dark red dots), 50% effort over 3474.2 km2 (light red dots), 25% effort over 6948.4 km2 (light blue dots), and 12.2% effort over 14240 km2 (dark blue dots). Note here when comparing total coral cover, that we are assuming coral cover is 100% of reef area.


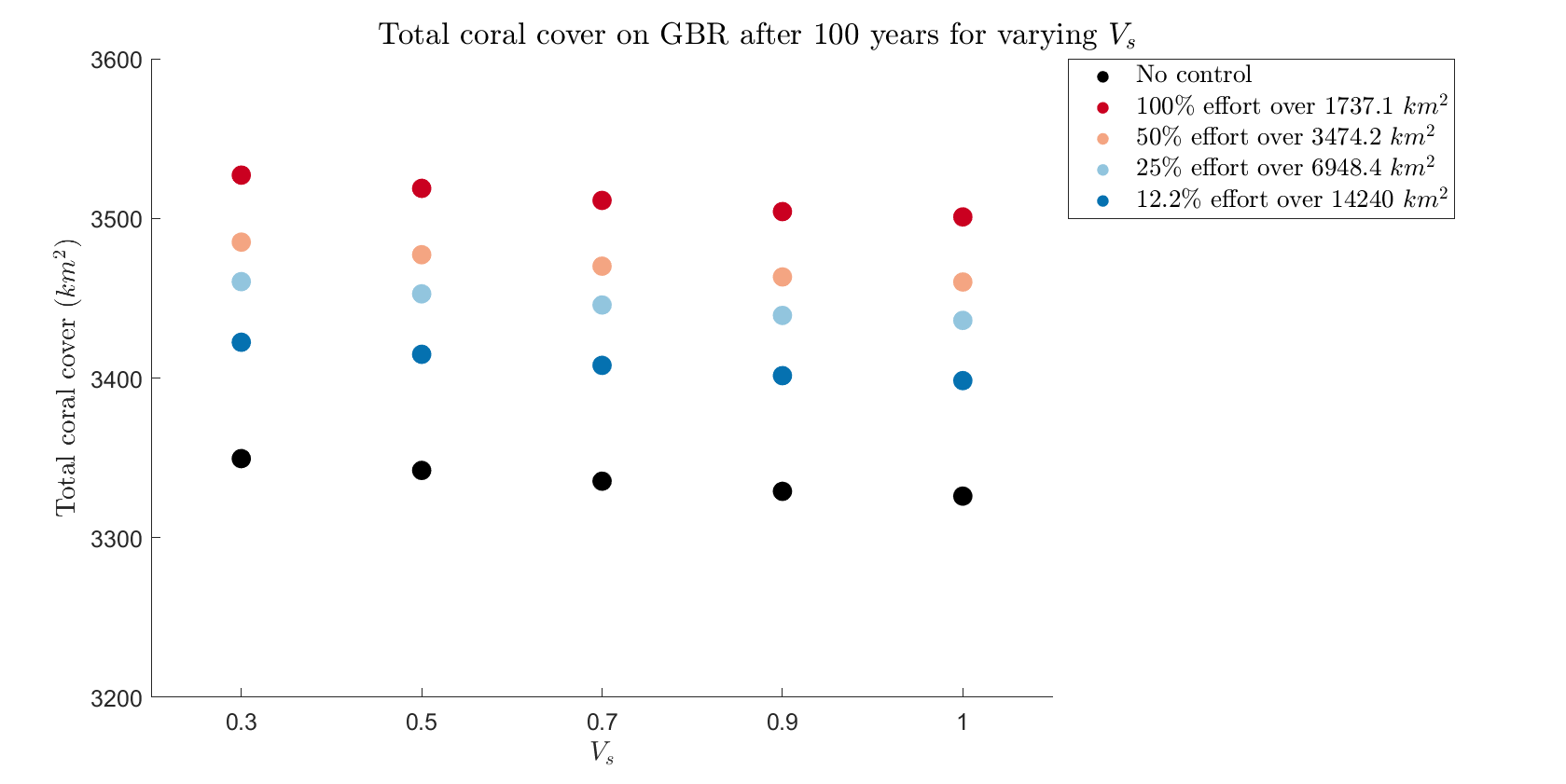


Figure 5 Total coral cover in km^2^ (sum of coral cover at each reef) on the Great Barrier Reef (GBR) after 100 years, for varying values of V_s_, the percentage of COTS larvae that survive once they settle at a reef, and varying control scenarios. The numbers tested for V_s_ are: 0.3, 0.5, 0.7, 0.9, and 1, and are shown on the x-axis. The control scenarios are: no control (black dots), 100% effort over 1737.1 km2 (dark red dots), 50% effort over 3474.2 km2 (light red dots), 25% effort over 6948.4 km^2^ (light blue dots), and 12.2% effort over 14240 km^2^ (dark blue dots). Note here when comparing total coral cover, that we are assuming coral cover is 100% of reef area.

1. **Sensitivity simulations**

We tested the results to sensitivity in the initial number of adult COTS to test the robustness of the impact of each control scenario on the GBR, with varying values of the initial number of adult COTS: 5, 10, 25, 50 and 100 adult COTS. Initialising the simulation with varying numbers of adult COTS had a very limited impact on results. Higher initial COTS abundances resulting in slightly lower total coral cover on the GBR (Figure 2). When initialised with 5 adult COTS at every reef on the initiation box, the total coral cover on the GBR with no control is 3364.38 $km^{2}$ whereas with 100 initial adult COTS, the total coral cover on the GBR with no control is 3329.03 $km^{2}$ (we are assuming coral cover is 100% of reef area). Thus a 20-fold increase in initial abundance had a 1% impact on consequences. The difference in initial COTS populations did not change the most effective control strategy.


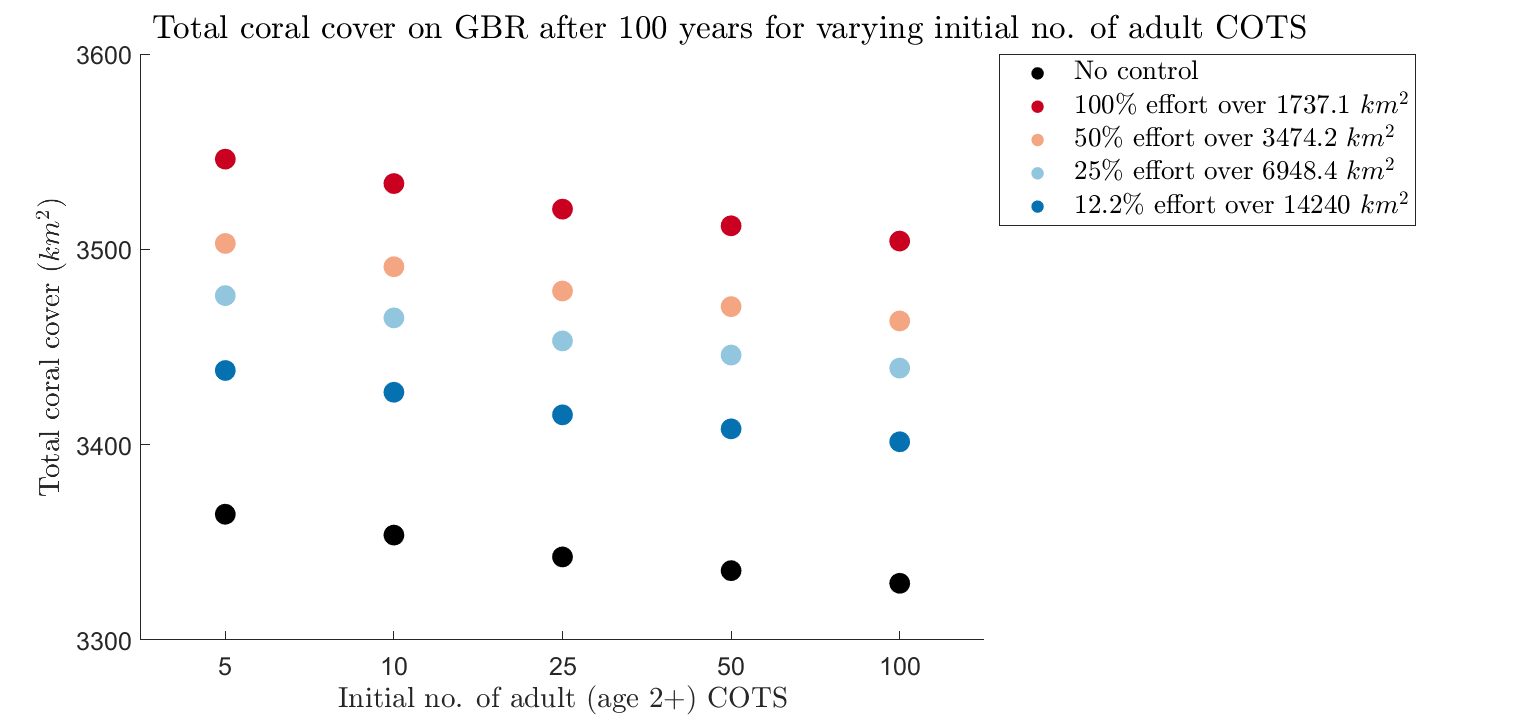


Figure 6: Total coral cover in $km^{2}$ (sum of coral cover at each reef) on the Great Barrier Reef (GBR) after 100 years, for varying initial numbers of adult COTS at each reef within the initiation box and varying control scenarios. The numbers of initial adult COTS are: 5, 10, 25, 50, and 100, and are shown on the x-axis. The control scenarios are: no control (black dots), 100% effort over 1737.1 $km^{2}$ (dark red dots), 50% effort over 3474.2 $km^{2}$ (light red dots), 25% effort over 6948.4 $km^{2}$ (light blue dots), and 12.2% effort over 14240 $km^{2}$ (dark blue dots). Note here when comparing total coral cover, that we are assuming coral cover is 100% of reef area.

**References**

1. Great Barrier Reef Marine Park Authority 1998. Great Barrier Reef Features (Version 1.4) [Dataset] 2164DB88-FD79-449E-920F-61C37ADE634B. Retrieved from <http://www.gbrmpa.gov.au/geoportal>.
